# DNA-Repair Gene Mutations Are Highly Prevalent in Circulating Tumour DNA from Multiple Myeloma Patients

**DOI:** 10.3390/cancers11070917

**Published:** 2019-06-29

**Authors:** Sridurga Mithraprabhu, Jay Hocking, Malarmathy Ramachandran, Kawa Choi, Daniela Klarica, Tiffany Khong, John Reynolds, Andrew Spencer

**Affiliations:** 1 Myeloma Research Group, Australian Centre for Blood Diseases, Alfred Hospital-Monash University, Melbourne 3004, Victoria, Australia; 2 Malignant Hematology and Stem Cell Transplantation, Alfred Hospital, Melbourne 3004, Victoria, Australia; 3 Epidemiology and Preventive Medicine, Alfred Health‐Monash University, Melbourne 3004, Australia; 4 Department of Clinical Hematology, Monash University, Clayton 3800, Victoria, Australia

**Keywords:** circulating tumour DNA, multiple myeloma, haematology, liquid biopsy, DNA-repair genes, *TP53*, RAS, prognosis

## Abstract

Mutational characterisation utilising plasma (PL)-derived circulating tumour DNA (ctDNA) in multiple myeloma (MM) has been recently described. Mutational analyses of paired bone marrow (BM) MM cell DNA and ctDNA from 76 patients (*n* = 24, new diagnosis (ND), *n* = 52, relapsed/refractory (RR)) for (ras/raf signaling pathway) and tumour protein p53 (*TP53)* mutations using the OnTarget™ Mutation Detection (OMD) platform was performed. The total number and proportions of mutations in each of the compartments (BM-specific, PL-specific or shared) was significantly higher in RR patients compared to ND patients (*p* = 0.0002 and *p* < 0.0001, respectively). Patients with > 2 mutations or > 1% fractional abundance (FA) in the PL had significantly shorter overall survival (OS) (*p* = 0.04 and *p* = 0.0006, respectively). Patients with PL-specific *TP53* mutations had significantly shorter OS compared to patients with no PL-*TP53* mutations (*p* = 0.003), while no differences were observed in patients with (K-ras) *KRAS* mutations. Targeted deep amplicon sequencing (TAS) of matched PL and BM samples from 36 MM patients for DNA-repair and *RAS-RAF* pathway genes found that DNA-repair genes were present at significantly higher levels in the PL when compared to *RAS-RAF* mutations (*p* = 0.0095). We conclude that ctDNA analysis identifies a higher prevalence of potentially actionable DNA-repair gene mutated subclones than BM analysis.

## 1. Introduction

Multiple myeloma (MM) is an incurable haematological malignancy characterised by multi-focal tumour deposits throughout the bone marrow (BM). The clinical outcome of MM is variable due to acquired mutations that contribute to disease progression and resistance to therapy. Several publications describing whole exome sequencing of BM have demonstrated the prevalence of mutations in the ras/raf signaling pathway (RAS/RAF), nuclear factor kappa-light-chain-enhancer of activated B cells (NF-KB), DNA-repair and cell cycle pathways, amongst others, in predominantly newly diagnosed (ND) MM patients [1,2,3,4]. More recently, associations between mutations in driver genes, primary translocation, hyperdiploidy and copy number variations have also been identified [5]. *RAS-RAF* pathway mutations have been identified in 40–50% of ND patients while tumour protein p53 (*TP53*) mutations are present in 5% to 6% of ND and between 21% to 26% in relapsed and/or refractory (RR) MM patients [2,3,4,6,7,8]. While the tumour genome for ND patients has been extensively described, this remains largely elusive for RR patients. Furthermore, the current practice for mutational characterisation in MM is through analysis of MM cell DNA sourced from BM biopsies and genetic information obtained from biopsies is confounded by the known inter and intra-clonal heterogeneity of the tumour(s), which we and others have shown is increased with disease progression [9,10,11]. Spatiotemporal analysis performed through comparison of BM aspirates and targeted biopsies from extramedullary disease sites, and multi-region sequencing in the same patient have indicated intra-patient heterogeneity [11,12,13,14]. While potentially informative, this approach is invasive, often not successful and subject to sampling bias. We have recently established an alternative approach that provides a more comprehensive picture of the genetic landscape of individual MM patients through the analysis of circulating cell-free tumour DNA (ctDNA) derived from the plasma (PL) [11,15]. Multi-convergence and a predominance in the RAS-MAPK pathway (69%) and the presence of *TP53* mutations in 27% of the RR patients was identified [15]. However, the modest patient numbers of our prior study limited our ability to identify any correlations between the PL and BM mutational landscape and either progression-free survival (PFS) or overall survival (OS). In this current study, MM mutational characterisation was performed on ctDNA and paired BM samples from 76 patients utilising the highly sensitive OnTarget^TM^ Mutation Detection (OMD) platform to determine the prognostic significance of ctDNA detectable *RAS-RAF* and *TP53* mutations. This platform has a sensitivity of 0.0001% and is specifically tailored to detect hot-spot mutations in k-ras (*KRAS*), n-ras (*NRAS*), b-raf (*BRAF*) and *TP53*. Results from this platform were validated in a less sensitive, albeit more comprehensive methodology, namely targeted deep amplicon sequencing (TAS). TAS of 36 contemporaneously sourced BM and PL samples was performed for *RAS-RAF* (*KRAS*, *NRAS* and *BRAF*) and DNA-repair genes (*TP53*, Ataxia telangiectasia mutated (*ATM*) and Ataxia telangiectasia and Rad3 related (*ATR*) that are known to be relevant to MM, revealing a predominance of DNA-repair genes alterations in the PL of RR patients.

## 2. Results

### 2.1. PL-Specific Mutations Characterise the Advanced MM Tumour Genome

**Figure 1:**
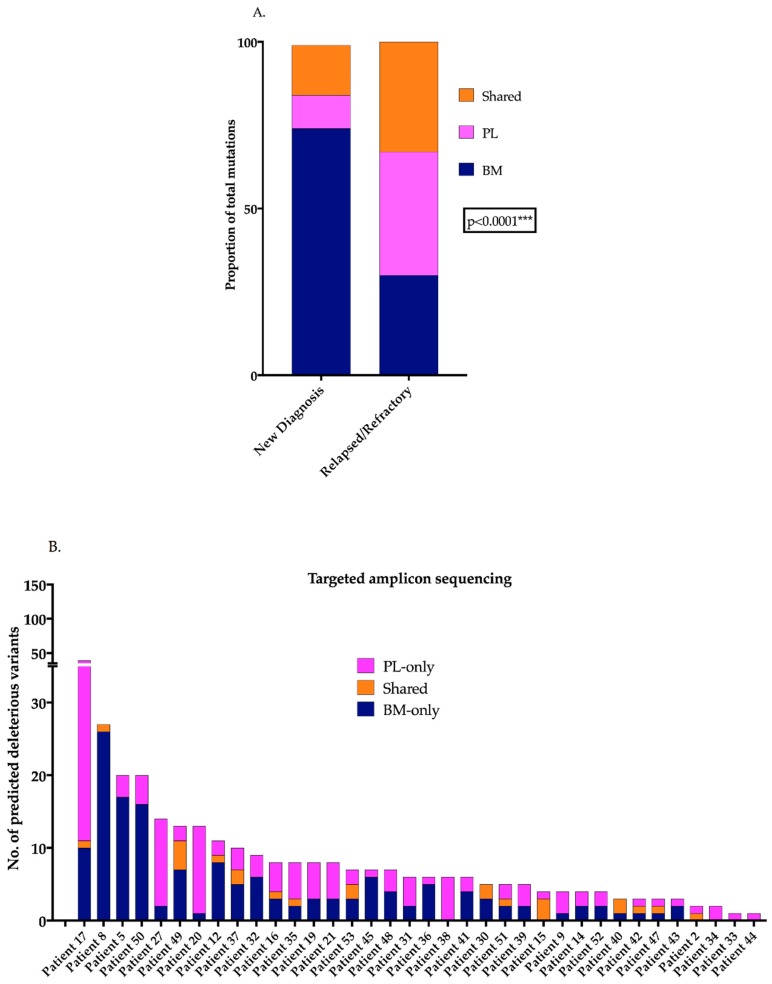
PL-exclusive mutations are present in MM patients (**A**) Column graph represents the proportion of mutations within the ND and RR patients (OMD) indicating the significant increase in the proportion of mutations detected in the PL-only in RR patients (Chi-square test, *** *p* < 0.0001) (**B**) Column graph represents the number of predicted deleterious and cancer driver variants in matched BM and PL samples obtained from 36 patients through TAS with 92% of patients harbouring variants present exclusively in the PL. PL: plasma, MM: multiple myeloma, ND: new diagnosis, RR: relapsed/refractory, OMD: OnTarget^TM^ mutation detection, TAS: targeted amplicon sequencing.

OMD of *KRAS*, *NRAS*, *BRAF* and *TP53* mutations in matched BM and PL from 76 MM patients indicated that RR patients (*n* = 52) had significantly more mutations in the plasma (PL) than ND patients (*n* = 24) (*p* = 0.0002, Chi-square test, data not shown). The mean number of total mutations per ND or RR patient was 1.6 or 2.9, respectively, and the mean number of PL-specific mutations per ND or RR patient was 0.19 or 0.94, respectively. The proportions of mutations within the BM and PL compartments were also significantly different, with RR patients demonstrating an increased mutational presence exclusive to the PL (*p* < 0.0001, Chi-square test, Figure 1A). Of the 76 matched patients, 21 (27.6%) of the patients had PL-specific mutations and 31 (40.8%) has BM-specific mutations for the genes tested (Table S1). Within this cohort, 36.5% of RR patients were found to have PL-specific mutations compared to only 8.3% of ND patients. Comprehensive TAS of 23 MM-specific genes in matched ctDNA and BM from 36 patients (*n* = 5 ND and *n* = 31 RR) confirmed the presence of PL-exclusive variants in 33/36 (91.7%) of patients (Figure 1B, Table S1). Additionally, all 5 ND patients (100%) and 28 RR patients (90.3%) had PL-exclusive mutations.

### 2.2. Patients with Higher Number and Tumour Burden of PL Mutations Have Significantly Shorter Survival

**Figure 2:**
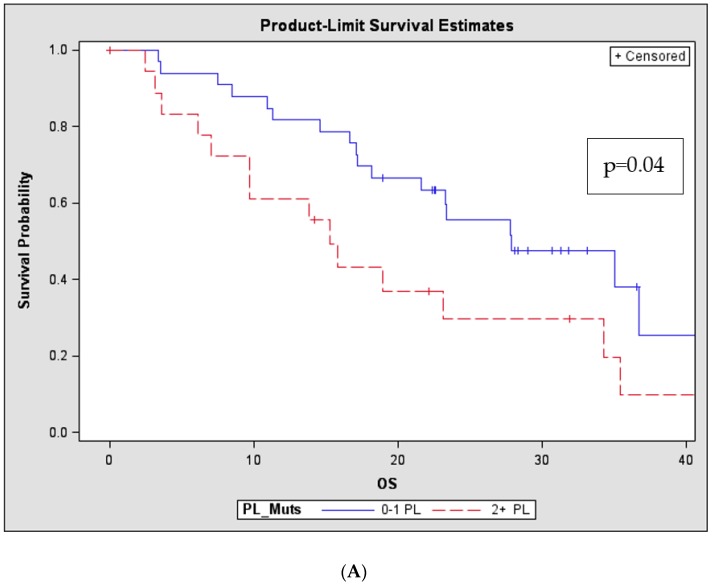

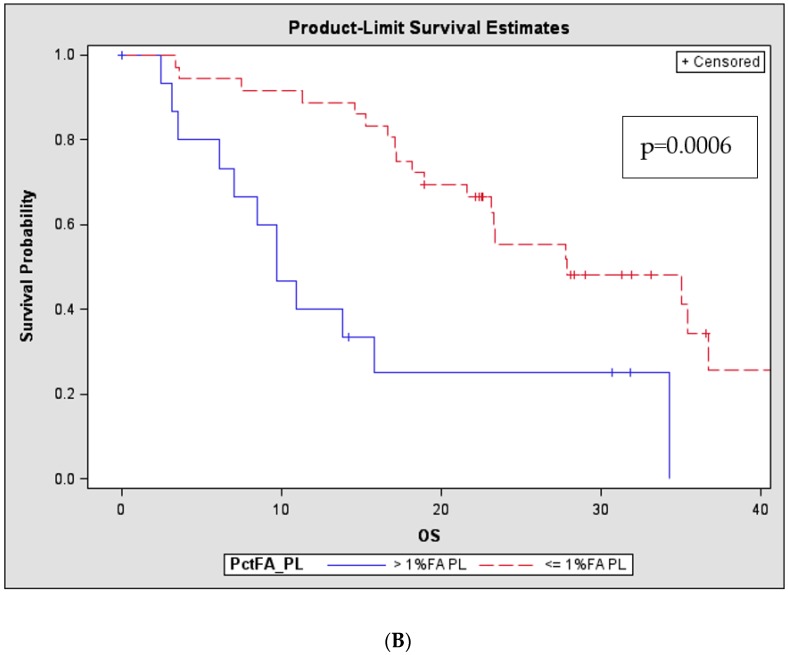
Number and mutational load of PL-mutations in advanced disease has prognostic implications in RR patients Kaplan-Meier (Product-Limit) OS curves and log-rank tests for RR patients (*n* = 52) based on the number and FA of mutations in the PL determined through OMD analyses (**A**) Patients with 2 + PL mutations had significantly shorter OS compared to patients with 0–1 PL mutations (*p* = 0.040) (**B**) The FA of the mutations represents the burden of the specific mutation in the designated compartment. Patients with >1% FA in the PL had significantly shorter OS compared to patients with lesser tumour burden in the PL (<1% FA; *p* < 0.001). (PL: plasma, RR: relapsed/refractory, OS: overall survival, FA: fractional abundance, OMD: OnTarget^TM^ mutation detection)

The potential prognostic significance of the number of detectable PL or BM mutations was then evaluated. As BM mutational presence is derived from enriched MM cells, the cut-off for the BM comparison was set at >2 or <2 mutations, whereas, since PL consists of a pool of both normal and tumour derived DNA, the comparison was set at >1 or <1 mutation. Log-rank tests revealed that in the RR patients, but not ND patients, the presence of >1 mutation in the PL had a significant impact on OS, conversely there was no impact related to the presence of >2 BM-detectable mutations (*p* = 0.04, Figure 2A and *p* = 0.41, respectively). Likewise, the “tumour burden” as defined by the fractional abundance (FA) of driver mutations in both the BM (patient grouped as >10% or <10%) and PL (patients grouped as >1% or <1%) was also evaluated for an association with patient outcome. Increased BM tumour burden was numerically associated with outcome, but this was not statistically significant (*p* = 0.058), in marked contrast, tumour burden as defined by the presence of FA > 1% in the PL of RR patients was strongly associated with inferior OS when compared to patients with < 1% FA in the PL (*p* = 0.0006, Figure 2B).

### 2.3. Clonal Mutations in the BM are More Likely to Be Present in the PL

**Figure 3:**
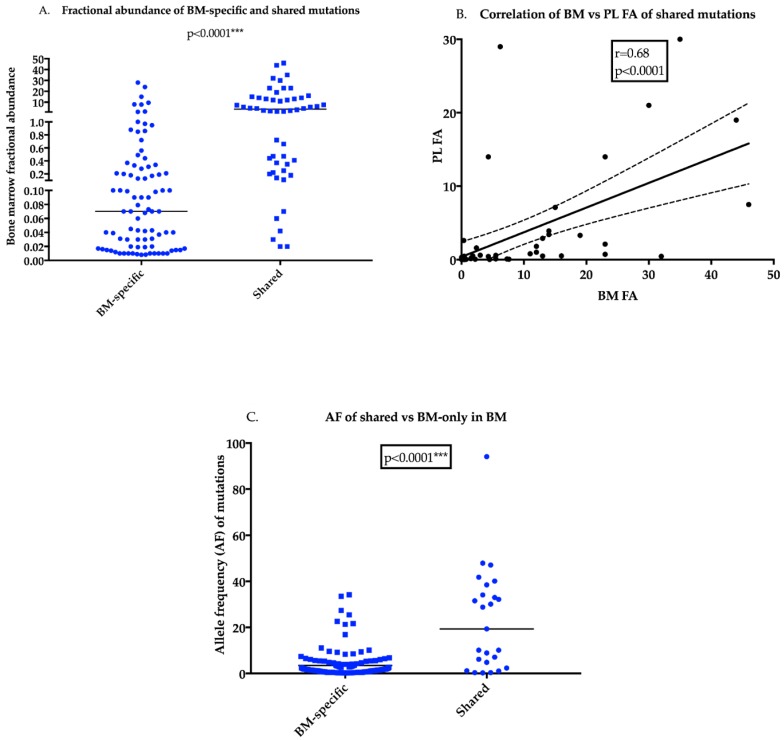
Dominant mutations in the BM have a higher likelihood of presence in PL (**A**) BM FA levels for two kinds of mutations (i) specific to the BM compartment versus (ii) present in both BM and PL compartments utilising a scatter dot-plot. Median FA levels are indicated by the black line derived from the OMD analyses. The median FA of shared mutations is significantly higher than FA of mutations detected in the BM alone (Mann Whitney *t*-test, *** *p* < 0.0001). (**B**) Correlation plot of BM AF and PL AF of shared mutations detected by OMD analyses indicates a moderate to strong association of BM AF with AF in the PL (*p* < 0.0001) (**C**) Scatter dot-plot with median levels represents a comparison of the AF of shared mutations and BM-only mutations derived from 36 matched BM and PL from MM patients utilising TAS. Results of the OMD were recapitulated in TAS indicating that shared mutations have a significantly higher BM AF (Mann Whitney *t*-test, *** *p* < 0.0001).(PL: plasma, BM: bone marrow, FA: fractional abundance, AF: allele frequency, TAS: targeted amplicon sequencing, OMD: OnTarget^TM^ mutation detection)

An analysis of the FA of OMD detected mutations in the BM comparing those mutations specific to the BM with those in both the BM and PL demonstrated significantly increased median FA levels for the shared mutations compared to BM-specific mutations (Figure 3A, *p* < 0.0001). Of the BM-specific mutations only 9/86 (or 10% of) had a FA > 1% whereas 32/52 (or 62% of) mutations present in both components had a FA > 1% in the BM. An XY-correlation plot for FA of BM vs. PL for shared mutations showed a 68% correlation (*p* < 0.0001, Figure 3B). TAS re-capitulated findings of the OMD with a significantly higher AF of shared mutations in the BM than BM-specific mutations (Figure 3C, *p* < 0.0001; Table S1)

### 2.4. TP53 Mutations are Frequently Detected in the Plasma of MM Patients

**Figure 4:**
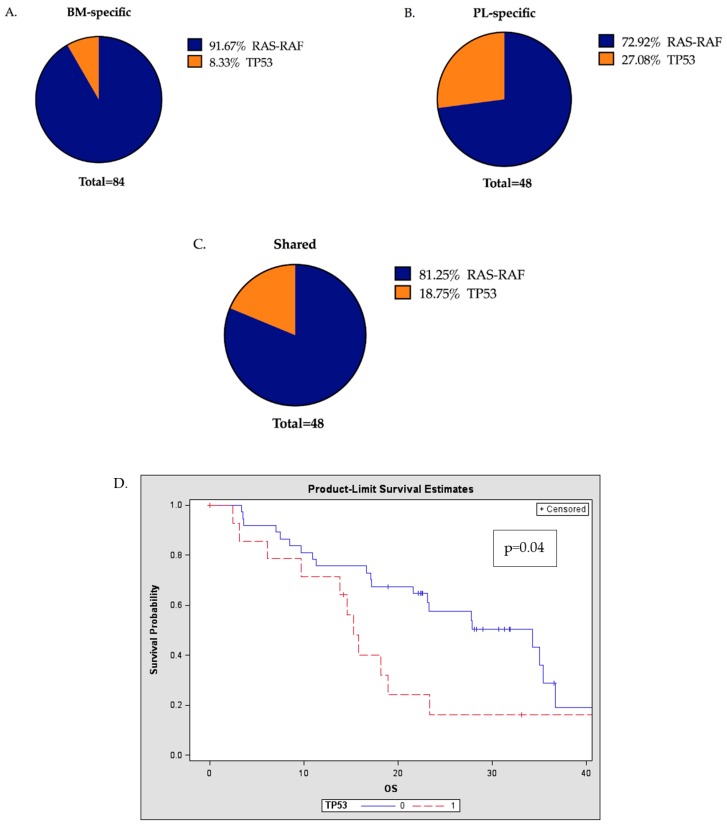

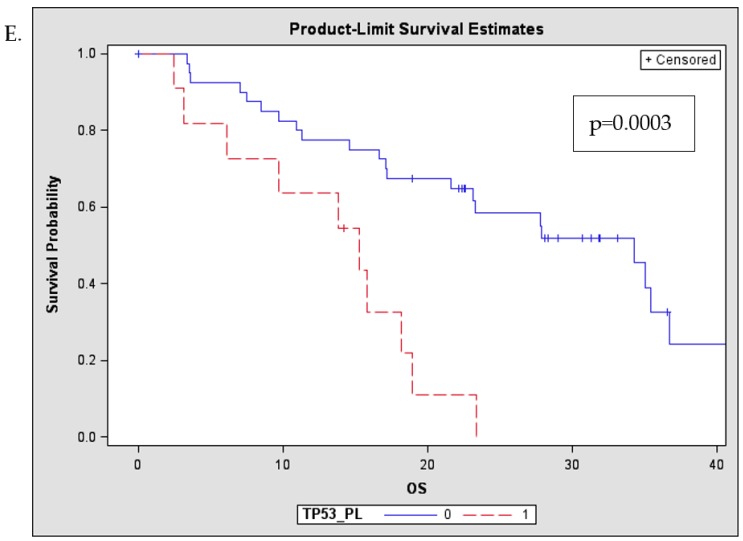
Presence of *TP53* mutations in the PL has a significant prognostic implication (**A**–**C**) OMD: Pie-charts represent the proportion of *RAS-RAF* and *TP53* mutations in BM-specific, PL-specific and shared mutations with a significant change in the proportions between the three populations (Chi-square, * *p* = 0.0159) (**D**) Kaplan-Meier (Product-Limit) OS plots and log-rank tests to compare RR patients with the presence (1 or more) or absence (0) of *TP53* mutations, indicating that *TP53* mutational presence either in the BM or PL is a significant prognostic factor (*p* = 0.04) (**E**) Patients with 1 or more *TP53* mutations in the PL had significantly shorter OS compared to patients with no *TP53* mutations in the PL (*p* = 0.003) (BM: bone marrow, PL: plasma, OS: overall survival, RR: relapsed/refractory)

Within the total pool of mutations detected, the proportion of mutations involving the *RAS-RAF* pathway or *TP53* were found to be significantly different when comparing those that were BM-specific, PL-specific or shared (BM and PL) (Figure 4A–C, *p* = 0.015, Chi-square). A higher proportion of *TP53* mutations was found in the PL-specific compartment compared to the BM (27% vs. 8%) and the presence of *TP53* mutations was also significantly more common in the RR patients compared to ND patients (*p* < 0.0001). Patients with *TP53* mutations were found to have significantly shorter OS compared to patients with no *TP53* mutations (*p* = 0.04, Figure 4D), and this adverse impact was more pronounced in the presence of PL detectable *TP53* mutations (*p* = 0.003, Figure 4E). However, there were no differences in the OS of patients with or without *KRAS* mutations in either the BM or PL (*p* = 0.67 and *p* = 0.47 respectively).

### 2.5. DNA-Repair Gene Variants are Present at Higher Levels in the Plasma of MM Patients

**Figure 5:**
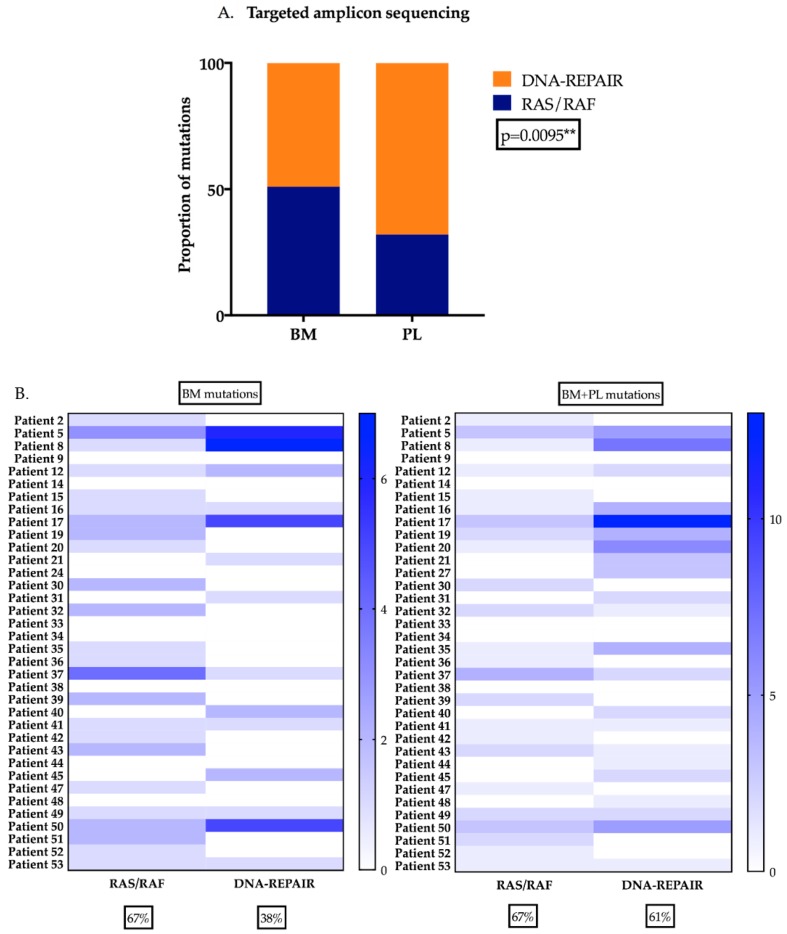

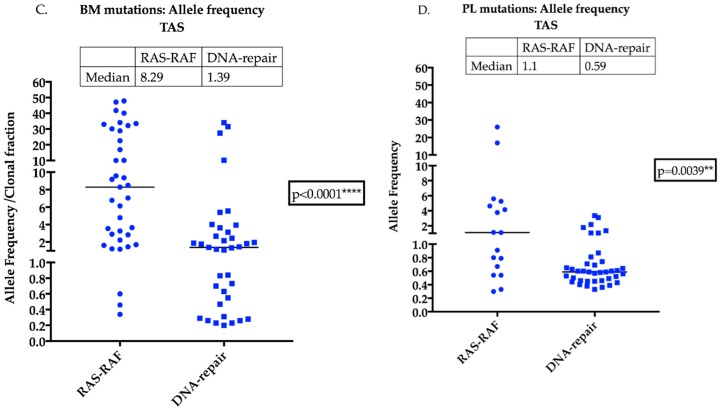
A higher prevalence of DNA-repair mutations in the PL of MM patients (**A**) TAS of DNA-repair genes (*ATM*, *ATR* and *TP53*) and *RAS-RAF* (*KRAS*, *NRAS* and *BRAF*) was performed on 36 matched contemporaneously obtained BM and PL samples from ND and RR patients. In the BM, the prevalence of predicted deleterious variants were present in approximately equal proportions from both pathways. The proportion of variants present in DNA-repair genes was significantly higher when compared to *RAS-RAF* pathway variants in the PL (Chi-square test, ** *p* = 0.0095) (**B**) Proportion of patients with *RAS-RAF* variants were found to be equally present when information from BM and PL is considered with >60% of patients harbouring variants. The number of patients with variants in DNA-repair pathway was higher when information from both BM and PL is considered. (**C**) AF of variants detected in the either *RAS-RAF* or DNA-repair pathway detected in the BM indicates that *RAS-RAF* pathway have a significantly higher median AF compared to DNA-repair (**D**) The PL AF of RAS-RAS and DNA-repair variants is significantly different although the difference is not as pronounced as in the case of the BM. (Pl: plasma, BM: bone marrow, AF: allele frequency).

Analysis of TAS results indicated that the prevalence of mutations in the DNA-repair pathway was significantly higher than *RAS-RAF* pathway mutations when information from either the BM or PL was evaluated (Chi-square test, *p* =0.0095, Figure 5A). Furthermore, when the proportion of patients harbouring *RAS-RAF* and/or DNA-repair pathway mutations in the BM or BM + PL was compared it demonstrated that *RAS-RAF* pathway mutations were of equivalent frequency (67% for both BM and PL), whereas for the DNA-repair genes the proportion increased from 38% (BM) to 61% (BM + PL) (Figure 5B). The median AF of mutations detected in the BM or PL when compared between *RAS-RAF* and DNA-repair revealed that the median AF of *RAS-RAF* pathway mutations was significantly higher, both in the BM and PL compared to DNA-repair gene mutations (*p* < 0.0001 and *p* = 0.0039 respectively, Figure 5C,D). Finally, 16% of the patients had mutations in DNA-repair genes exclusively in the PL compared to only 2.5% of *RAS-RAF* pathway genes mutations (Table S1, data not shown).

## 3. Discussion

Our study has revealed the utility of PL ctDNA analyses for comprehensive mutational characterisation and identification of mutations driving disease progression in MM patients. Data presented here has established that a higher number of detectable PL mutations is associated with significantly inferior OS, consistent with the hypothesis that increased spatial genetic heterogeneity represents an adverse biological state in MM, with a greater likelihood of resistance to therapy. Consequently, characterising such heterogeneity may be of particular importance in instances where a resistance mechanism is being sought to explain why some patients, particularly RR patients, are unresponsive to therapy. For example, differences in responsiveness to therapy may be able to be defined at the mutational level, whereby resistance to a specific drug is being conferred by the presence of one or more driver mutations in oncogenes and/or tumour suppressors with these lesions being more readily identifiable with liquid biopsy than single-site BM biopsy approaches. Adopting such a strategy to characterise the tumour-specific mutational landscape would provide a more informative avenue for personalised or biomarker-based therapy in MM, a disease where the genomic landscape of each individual tumour is heterogeneous and will change over time due to the selective pressures imposed on MM cells during therapy.

PL ctDNA mutational analyses in ND showed that the number of mutations detected in the PL with OMD was only 8.3%, while with TAS, all 5 ND patients had PL-mutations detected. This suggests that strategies targeting increased numbers of mutations are more likely to identify spatial heterogeneity even in ND patients, and this heterogeneity becomes more diverse for RR patients. This confirms the potential of ctDNA analysis for the genetic characteristation of MM patients at both presentation and relapse. Importantly, we have confirmed that the number of PL-detectable mutations and the tumour burden in the spatial compartment is a prognostic factor for RR as demonstrated here and in a second and independent cohort of RR patients that we have recently described [16]. Comparison of the FA (from OMD) and the AF (from TAS) between co-existing BM and PL mutations with BM-only mutations indicated that a higher FA/AF of BM clones was associated with an increased likelihood of these BM mutated clones also being detected in the PL, consistent with the hypothesis that mutations evident in the PL reflect the predominant mutations driving disease progression originating from all focal sites, both intramedullary and extramedullary. Therefore, ctDNA analysis may provide critical clues about the predominant driver mutation orchestrating resistance to therapy in MM. In other cancers, ctDNA analysis has been utilised to predict therapeutic response, to identify mutations associated with the emergence of drug resistance [17,18,19,20,21] and as a biomarker in the clinical setting for treatment-decision making, specifically in cases wherein invasive diagnostic procedures are not feasible [22,23]. Such applications are also applicable to MM.

Our evaluation of the mutations present in the *RAS-RAF* and *TP53*/DNA-repair pathways with both OMD and TAS has established the significance of each of these pathways in the spatial genome of advanced patients. PL has a higher proportion of *TP53* mutations than BM, signifying that these mutations are predominant in the spatial genome. Therefore, information from both BM and PL would provide a more holistic picture of the tumour genome, with our data demonstrating that a proportion of prognostically significant *TP53* mutations will likely have been undetected in prior whole exome sequencing/single-site BM biopsy studies. Additionally, RR patients have a high prevalence of DNA-repair gene variants, indicating that these mutations may play a critical role in clonal evolution that occurs during relapse. Moreover, comparison of the AF of mutations in the BM and PL involving the *RAS-RAF* and DNA-repair pathways indicated that although the *RAS-RAF* pathway mutations appear to be dominant locally, the DNA-repair gene variants have a more predominant presence in the PL, suggesting that these variants are more likely to arise from multiple focal sites. Additionally, it could also be that the RAS mutations are ancestral to the DNA-repair gene mutations and are relatively *sub-clonal* when compared to RAS mutations.

Our data demonstrate that the presence of *TP53* mutations in the PL is an adverse prognostic factor. This result recapitulates that seen in previously published studies where ND patients harbouring mono or bi-allelic inactivation of *TP53* [4] or alteration in *TP53* [6] have been shown to have an inferior prognosis. Likewise, longitudinal comparison of patients at presentation and relapse has revealed that bi-allelic events i.e., deletion in 17p/*TP53* mutation or deletion *TP53* portends a negative outcome after relapse [7]. *ATM*, *ATR* and *TP53* are tumour suppressors and key mediators of the DNA damage response (DDR) and therefore, mutation or loss of function of these specific targets may lead to genomic instability that can in turn can accelerate drug resistance. Loss of heterozygosity (LOH) induced by homologous recombination deficiency (HRD) resulting from DNA-repair pathway gene mutations increases as the disease advances, and RR patients with high levels of HRD-LOH have a worse outcome [24]. Consequently, regulators of DDR are attractive targets for therapy and a number of small-molecule inhibitors against these have been tested in MM [25]. Published studies also report that defects in *ATM* or *ATR* signaling can be synthetically lethal with PARP inhibition [26], and although pre-clinical evidence for the use of PARP inhibitors in MM exists, a biomarker-based approach has not been evaluated clinically [27,28].

## 4. Materials and Methods

### 4.1. Patient Population

BM and matched peripheral blood (PB) samples were collected from MM patients following written informed consent as per an Alfred Hospital Human Research and Ethics Committee-approved protocol (29/05, 5th August 2015). ND or RR patients with active disease and >5% disease burden on BM biopsy were studied. OMD analyses were performed on 76 matched BM and PL samples including *n* = 24 ND and *n* = 52 RR patients (48 described previously in [15] with an additional 28 included in this study, Table S1). Furthermore, TAS was performed on an additional and independent cohort of 36 matched BM and PL samples, along with PBMC germ line controls (*n* = 5 ND, *n* = 31 RR patients-Table S1).

### 4.2. Peripheral Blood (PB) Collection and Processing

Peripheral blood PL was collected into Streck BCT DNA (tubes Streck, cLa Vista, NE, USA). Immediately upon sample collection, the tubes were inverted to mix the blood with the preservative in the collection tube. PL was separated from PB through centrifugation at 820 g for 10 min within 24 h of sample collection. Supernatant was collected without disturbing the cellular layer and centrifuged again at 16,000 g for 10 min to remove any residual cellular debris and stored at −80 °C in 1 mL aliquots for long-term storage until further analyses.

### 4.3. Isolation of Mononuclear Cells and MM Cells

Peripheral blood mononuclear cells (PBMC) and BM aspirates were collected into EDTA tubes and subjected to ficoll isolation of bone marrow mononuclear cells (BMMNC) and PBMC, respectively, as previously described [29]. PBMC were snap frozen as cell pellets and stored at −80 °C until further analysis. MM cell proportions in the BMMNC samples were measured with flow cytometry and MM cells were subsequently isolated using CD138+ magnetic beads (Miltenyi, Bergisch Gladbach, Germany [29]) then snap frozen and stored at −80 °C.

### 4.4. Genomic DNA Extraction From BM Aspirates/ Trephines

Frozen pellets of CD138+ MM or PBMC cells were subjected to DNA extraction using the QIAGEN Blood DNeasy extraction kit (QIAGEN, Hilden, Germany). following manufacturer’s instructions. For OMD analyses, for 6 of the patients, triplicate10-micron sections of formalin fixed paraffin-embedded BM trephine was used as the source of DNA utilising the High Pure FFPET DNA isolation kit (Roche, Basel, Switzerland) according to the manufacturer’s instructions. All DNA was quantified with QUBIT Fluorometer 2.0 (Thermo Fisher Scientific, Waltham, MA, USA).

### 4.5. OnTarget^TM^ Mutation Detection (OMD) Platform

OMD is an assay developed for the detection of ctDNA across a panel of cancer-related mutations with high sensitivity and specificity based upon sequence-specific synchronous coefficient of drag alteration (SCODA) technology, which enables efficient enrichment of mutant DNA from PL. The OMD platform chosen had mutations in *KRAS*, *NRAS*, *BRAF* and *TP53*, in addition to other solid cancer relevant genes. Of the 76 matched PL and BM samples, OMD had been previously undertaken for *n* = 48 samples [15]. A further 28 matched samples were added to this cohort also utilsing the OMD platform (Boreal Genomics, Vancouver, BC, Canada). The methods for OMD sample extraction, quantification, processing, mutation enrichment, MiSeq library preparation, sequencing and data analysis are as described previously [30]. The number of mutations in each compartment, the mutational fractional abundance (FA) (defined as the ratio of mutant copies to total input genome copies) and the type of mutation was correlated to both PFS and OS measured from the date of commencement of the first subsequent line of therapy after mutational characterisation.

### 4.6. Targeted amplicon sequencing

TAS was performed using a 23-gene customised panel including *KRAS*, *NRAS*, *BRAF*, *ATM*, *ATR* and *TP53* (all MM-relevant genes) at QIAGEN Service Core, Hilden, Germany. cfDNA was isolated from 5 mL aliquots of plasma using the QIAseq cfDNA All-in-One Kit. Isolated DNA was quantified with the Quant-iT dsDNA High-Sensitivity Assay Kit (Thermo Fisher Scientific, Waltham, MA, USA. cat.no. Q32851) or the Quant-iT dsDNA BR Assay Kit (Molecular Probe/Life Technologies, cat.no. Q32853). The QIAseq Targeted DNA Panel Kit was used for library generation and target enrichment. Fragment size distribution of the libraries was determined with an Agilent Bioanalyzer using a DNA 7500 chip (Agilent Technologies, Santa Clara, CA, USA), cat.no. 5067-1506). Library quantification was performed using the KAPA Library Quant Illumina Kit (Roche Holding, Basel, Switzerland, 07-KK4822-01). Indexed sample libraries were equimolarly pooled and sequenced on an Illumina NextSeq sequencer using a NextSeq 500 High Output v2 Kit (300 cycles) (Illumina, San Diego, CA, USA).

The PL samples were sequenced at an average read depth of 36,509 fold with an average molecular depth of 2021 and each molecular tag was found on an average of 10.8 fragments. The BM and germ line samples were sequenced at an average read depth of 20,246 fold, molecular tag depth of 3882 and each molecular tag was found on an average of 26.97 fragments. Data analysis including alignment to reference genome and variant calling was carried out using QIAGEN’s online QIAseq Targeted DNA online portal. Variant allele frequency or allele frequency (AF), defined as the relative frequency of an allele at a particular locus and expressed as a fraction or percentage, was derived for each sample set. Ingenuity variant analysis (IVA) software was utilised for variant annotation and pathway enrichment analysis. SNVs with a depth of coverage < 20 in tumour or PL samples and failed upstream filtering were excluded. Variants outside the top 5.0% most exonically variable 100base windows in healthy public genomes (1000 genomes) and outside the top 1.0% most exonically variable genes in healthy public genomes (1000 genomes) were included. Excluded variants were such that were observed with an AF greater than or equal to 3.0% of the genomes in the 1000 genomes project OR greater than or equal to 3.0% of the NHLBI ESP exomes (All) OR greater than or equal to 3.0% of the AFC Frequency OR greater than or equal to 3.0% of the ExAC Frequency OR greater than or equal to 3.0% of the gnomAD Frequency OR Filter variants unless it was an established pathogenic common variant. The default filter settings on IVA for “predicted deleterious and cancer driver variants” were employed. SNVs and INDELS appearing in the germ line control were excluded utilising the “tumour-specific variants” setting.

### 4.7. Statistical analyses

Statistical analyses were performed using GraphPad Prism 7.0f (GraphPad, San Diego, CA, USA) and SAS 9.4.(SAS, Cary, NC, USA). Detailed descriptions are provided within the results and figure legends.

## 5. Conclusions

In conclusion, our findings demonstrate that PL ctDNA analysis may provide an accessible medium for disease prognostication in RR and enhance the opportunity for the utilization of targeted therapies, particularly via the exploitation of identified alterations in DNA-repair genes. Identifying patients likely to respond to PARP inhibition based on the presence of one or more alterations in the DNA-repair genes would be advantageous clinically, as genomic instability is more prevalent in advanced and high-risk disease where therapeutic options remain limited.

## Supplementary Materials

The following are available online at https://www.mdpi.com/2072-6694/11/7/917/s1, Table S1: List of mutations detected by OMD and TAS in MM patients

## Abbreviation

MM	multiple myeloma
BM	bone marrow
PL	plasma
ctDNA	cell free tumour DNA
ND	newly diagnosed
RR	relapsed and/or refractory
OMD	OnTargetTM mutation detection platform
TAS	targeted amplicon sequencing
FA	fractional abundance
PFS	progression-free survival
OS	overall survival
AF	allele frequency
